# Characteristics and Distribution of Phosphorus in Surface Sediments of Limnetic Ecosystem in Eastern China

**DOI:** 10.1371/journal.pone.0156488

**Published:** 2016-06-09

**Authors:** Wenqiang Zhang, Xin Jin, Xiaolei Zhu, Baoqing Shan

**Affiliations:** State Key Laboratory on Environmental Aquatic Chemistry, Research Center for Eco-Environmental Science, Chinese Academy of Science, Beijing, 100085, P.R. China; NERC Centre for Ecology & Hydrology, UNITED KINGDOM

## Abstract

Phosphorus (P) is an essential nutrient for aquatic organisms; however, excessive P inflow to limnetic ecosystems can induce eutrophication. P concentrations in the rivers, wetlands and lakes of Eastern China have been amplified by fertilizer and sewage inputs associated with the development of industry and agriculture. Yet, knowledge of the distribution and speciation of P is lacking at the regional scale. We determined the distribution and speciation of P in limnetic ecosystems in Eastern China using Standards, Measurements and Testing (SMT) and phosphorus nuclear magnetic resonance (^31^P-NMR). The results indicate that P pollution in surface sediments was serious. Inorganic P (Pi) was the primary drive of variation in total P (TP) among different river systems, and Pi accounted for 71% to 90% of TP in surface sediment in Eastern China. Also, the concentrations of TP and Pi varied among watersheds and Pi primarily drove the variation in TP in different watersheds. Sediments less than 10-cm deep served as the main P reservoir. Environmental factors affect the speciation and origin of P. NaOH-Pi, HCl-Pi and organic P (Po) were related to pH accordingly at the regional scale. The physicochemical properties of sediments from different limnetic ecosystems affect the P speciation. HCl-Pi was higher in wetland sediments than in riverine and lake sediments in Eastern China. Conversely, NaOH-Pi was lowest in wetland sediments. Total Po concentration was lower in riverine sediments than in other sediments, but Mono-P was higher, with an average concentration of 48 mg kg^−1^. Diesters-P was highest in lake sediments. By revealing the regional distribution of TP, Pi and Po, this study will support eutrophication management in Eastern China.

## Introduction

Phosphorus (P) is an essential element for all organisms, and P availability can impact the rate of primary production in aquatic ecosystems [1]. Excessive P in the aquatic environment, however, can cause eutrophication [2]. Since the middle of the 20^th^ century, increasing application of P fertilizers has created a one-way flow of P from bedrock to farms and into rivers, lakes, wetlands and oceans that has dramatically influenced aquatic ecosystems worldwide [3].

In the last two decades, China has the booming economy around the world with GDP growth of nearly 8% annually [4]. During this time, human agricultural and industrial activities have greatly increased P generation and consumption, hence accelerating the rate of P bioactivity [5]. Particularly in Eastern China, intensified agriculture has been associated with the excessive application of fertilizers that research suggests only 10–20% of the P applied is used by the first crop cycle [6,7]. For example, in the year 2000, the application of P fertilizer to wheat crops exceeded 30 kg ha^–1^, which was higher than that in sub-Saharan Africa, central Asia or Latin America, where P loads were 0–5 kg ha^–1^ [8]. In fact, the Bohai Sea Economic Rim, Yangtze River Delta Economic Rim and Pearl River Delta Economic Rim, the three most densely populated and industrialized zones in China, are all located in Eastern China. The rapid population growth, industrial development and increased agricultural production have led to amplified inflow of untreated sewage and rubbish into the environment. The resulting P loads in the watershed greatly increase the risk of eutrophication in surrounding rivers, lakes and marine systems [9]. Additionally, the risk of eutrophication in wetland sediments is also high [10]. In the Pearl River estuary wetlands, P concentrations ranged from 648.9 to 1064.0 mg kg^−1^ in the surface sediment. Average total P (TP) concentrations of 648 mg kg^−1^ in Taihu Lake sediment (18 sites) and more than 1800 mg kg^−1^ in Meiliang Bay indicate that the sediment can be a potential origin of P for the water column and can contribute to eutrophication [11]. P stored in the sediment can not only induce environmental problems, but also disrupt the P biogeochemical cycle, causing new challenges for the sustainability of finite P resources [7].

The effects that anthropogenic P loading will have on the environment, however, depend on the uniformity of the P concentration, the relationship between different P speciation and environmental factors and the distribution of P among different ecosystems. We investigated the distribution of P speciation in different limnetic systems using SMT fractionations and solution ^31^P-NMR to analyze the P characteristics in the surface sediment of rivers, wetlands and lakes in Eastern China. We also examined the relationship between P speciation and environmental factors to provide a basis for developing mitigation strategies and management policies for P in a developing country.

## Materials and Methods

### Site description and sampling

**Fig 1:**
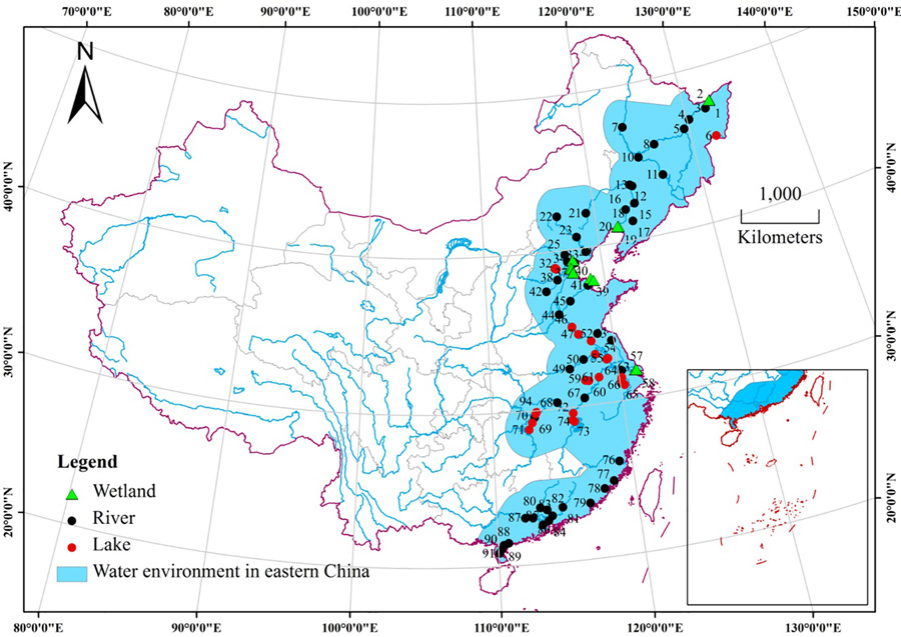
Location of the 89 sampling sites in Eastern China. Sites were distributed throughout Heilongjiang, Liaoning, Hebei, Shandong, Jiangsu, Anhui, Hubei and Guangdong provinces, as well as Beijing, Tianjin and Shanghai municipalities. River, wetland and lake systems, prominent ecosystems in the Eastern China, are represented.

To obtain representative sediment samples, sediments were collected from 89 sampling sites in Eastern China in September 2013 according to the watershed and the level of pollution (Fig 1 and S1 Fig). Sampling sites were located on rivers, wetlands and lakes from the Songhuajiang River to the Zhujiang River (S1 Table). All sampling sites were publically owned, and there were no endangered or protected species in the study area; thus no permits were required for sampling. At each site, three surface sediment samples (~5 cm deep) were collected using a Peterson grab sampler at locations at least 500 m apart. Subsamples from a site were pooled and homogenized to obtain a representative sample. Pooled samples were kept below 0°C in sealed plastic bags in a cooler inside a car. After arrived at the laboratory, samples were freeze-dried at −50°C in FD-1 freeze-dryers. Dried samples were ground and sieved with a 100-mesh sieve. A quarter of each sample was saved as the representative sample, and then samples were stored in sealed plastic bags and stored at room temperature until analysis.

### Sediment properties and SMT fractionation

Sediment pH was determined on sediments suspended in deionized water at a ratio of 1:2.5 [12]. Total carbon (TC) and total nitrogen (TN) were detected by elemental analyzer (Elementar, German, Vario EL III). Organic matter (OM) in sediments was determined by loss of ignition (LOI) at 550°C for 4 h [13].

Using the SMT protocol [14,15], sediment P content was classified into TP, inorganic P (Pi), organic P (Po), P associated with Ca (HCl-Pi) and P bound to Fe, Al and Mn oxides and hydroxides (NaOH-Pi). For TP analysis, sediment samples were extracted with 3.5 M KCl for 16 h with shaking at room temperature (~25°C), calcined for 3 h at 450°C, and TP was measured in the resulting extract. For Po analysis, sediments were extracted with 1 M HCl for 16 h with shaking, the residue was calcined for 3 h at 450°C and extracted with 1M HCl, and Po was determined on the resulting extract. Pi was analyzed in two ways. First, sediments were extracted with 1M NaOH for 16 h with shaking and centrifuged. After extraction with 3.5 M HCl and centrifugation, the extract was used for NaOH-Pi determination. The residue was extracted with 1 M HCl, and HCl-Pi was measured in the resulting extract.

### NaOH-EDTA extraction and ^31^P-NMR analysis

**Fig 2:**
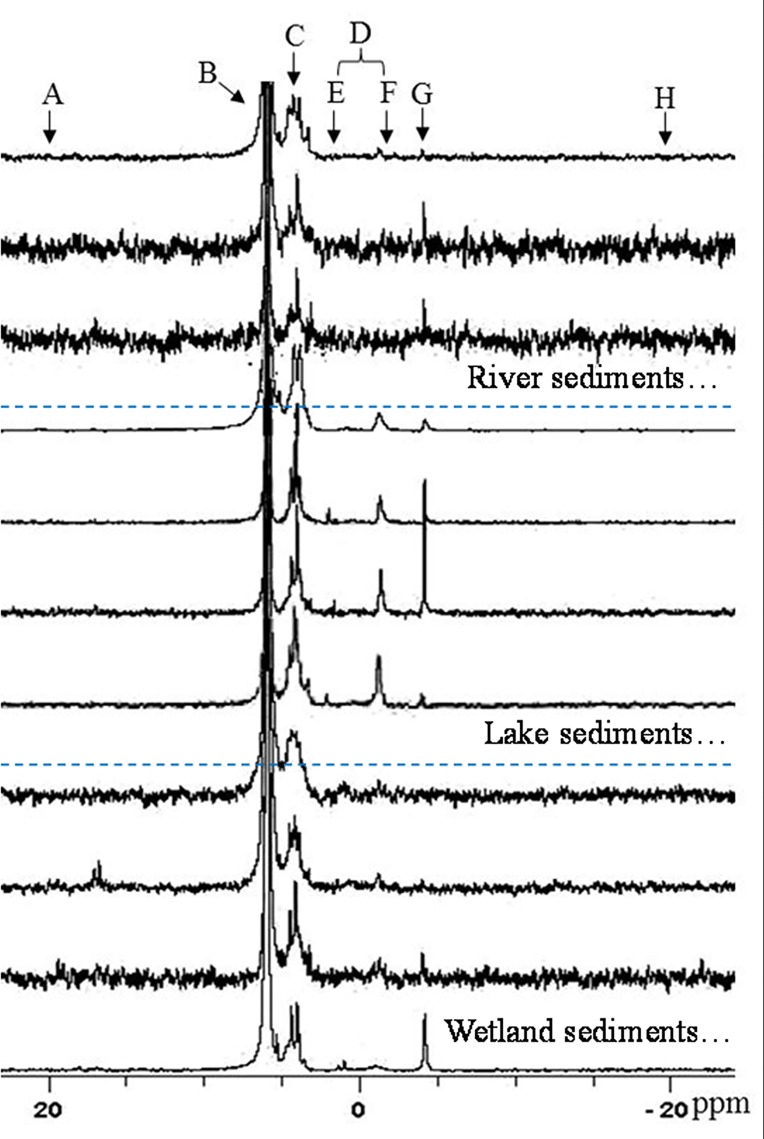
Partial ^31^P-NMR spectra of NaOH-EDTA extracts of the surface sediments in freshwater ecosystems of Eastern China. A: phosphonate; B: orthophosphate; C: orthophosphate monoesters; D: orthophosphate diesters; E: phospholipids; F: deoxyribonucleic acids; G: pyrophosphates; H: polyphosphates.

For solution ^31^P-NMR analysis, sediment samples (3.0 g) were extracted for 16 h at room temperature with 30 mL of a solution composed of 0.05 mol L^−1^ EDTA + 0.25 mol L^−1^ NaOH [16]. The pre-concentration of NaOH-EDTA extracts include rotary evaporation and freeze-drying. Researchers demonstrated the two methods with their shortcoming and advantage [17–19]. We also assessed the two methods [20] and chosen the freeze-drying as the pre-concentration method in this study. The solution was frozen and lyophilized for several days before analysis by ^31^P-NMR spectroscopy. Just before analysis, lyophilized NaOH-EDTA extracts (300 mg) were re-dissolved in 0.6 mL D_2_O and 0.1 mL 10 mol L^−1^ NaOH, ultrasonicated for 30 min and equilibrated for 5 min. Then 2% (v/v) 0.11 M NaHCO_3_ + 0.11 M Na_2_S_2_O_4_ (BD) was added to the extracts to reduce paramagnetic ion (Fe^3+^, Mn^2+^) interference [21,22]. The supernatants were centrifuged for 15 min at 14000 rpm and transferred to 5-mm NMR tubes. Solution ^31^P-NMR spectra were obtained using a Bruker 400 MHz spectrometer (Bruker, Billerica, MA, USA) operating at 129.53 MHz at 25°C. We used a 90° observation pulse with a relaxation delay of 3 s and an acquisition time of 0.6 s. Although the T1 relaxation times were not explicitly analyzed in this study, a T1 relaxation time of 3 s is considered adequate to obtain quantitative spectra for a variety of P forms from most samples (80%) analyzed for Mn and Fe [17,23]. Spectra were collected after 3,000–20,000 scans, depending on the concentration of Po. Chemical shifts were recorded relative to an 85% H_3_PO_4_ standard (δ = 0 ppm). Signals were assigned to P species based on data in the literature [24,25]. Peak assignments were made using ^31^P-NMR chemical shifts of phosphonate (phon-P: 12–23 ppm), orthophosphate (ortho-P: 6–7 ppm), orthophosphate monoesters (mono-P: 4–6 ppm), phospholipids (lipids-P: 1–3 ppm), deoxyribonucleic acid phosphorus (DNA-P: 0 ppm), pyrophosphate (pyro-P: -3.5– -4.5 ppm) and polyphosphates (- 20 ppm). Spectral processing was carried out using NMR Utility Transform Software (NUTS) for Windows (2010 edition Acorn NMR; Livermore, CA, USA). Using the area of each P species, the contribution of each P compound group was calculated relative to that of TP using NaOH-EDTA extraction and the molybdenum blue method (Fig 2) [26,27]. Briefly, NaOH-EDTA extracts were diluted by a factor of 50 to 100, digested by autoclaving with persulfate solution and analyzed spectrophotometrically as orthophosphate using the molybdenum blue method.

### Statistical analysis

Data were analyzed using OriginPro 8.0 for Windows. A biplot was constructed using SAS for Windows 9.2 (SAS Institute, Inc., Cary, NC, USA). Vector length in a biplot indicates the degree to which the variable represents the environmental factors. The angle between two vectors indicates the relationship between them.

## Results

### Phosphorus analysis by SMT

The concentration of TP ranged from 313.98 to 1224.45 mg kg^-1^ (average concentration of different river systems). The TP concentrations in most of the sampling points were higher than 500 mg kg^-1^. Pi was the main component in TP, with the concentration ranged from 243.58 to 1097.13 mg kg^-1^, and the percentage of Pi to TP ranged from 71.98 to 90.25%. For Pi, the HCl-Pi was much higher than NaOH-Pi. HCl-Pi was the main component of Pi, the rate of HCl-Pi to NaOH-Pi ranged from 0.59 to 15.62. The highest and lowest rates were at Yellow River (15.62) and Pearl River (0.59), respectively.

### Organic phosphorus components by ^31^P-NMR analysis

Three classes of Po compounds and one Po compound were detected in sediments using ^31^P-NMR: Phon-P, Mono-P, Lipids-P and DNA-P. Mono-P was the main components of Po in different river systems, which ranged from 22.22 to 118.45 mg kg^-1^. DNA-P was the second main component of Po, which ranged from 1.04 to 19.52 mg kg^-1^. The Phon-P and Lipids-P were not detected in all rivers. Phon-P was not detected in Yellow River and Huaihe River, and the Phon-P ranged from 0.38 to 6.81 mg kg^-1^. Lipids-P was not detected in Mindongnan Rivers, and the concentration of Lipids-P in another river system ranged from 0.71 to 12.63 mg kg^-1^.

#### Physicochemical property of the surface sediment

For the chemical-physical properties of sediments in whole sampling sites, the pH ranged from 4.56 to 8.94, the average concentration was 7.70; the LOI ranged from 0.54 to 16.70, the average concentration was 5.41; the TN and TC ranged from 0.01% to 0.98% and 0.12% to 13.62%, respectively. The TC/TN ranged from 6.14 to 107.78, the average concentration was 17.23. For different freshwater systems, the average pH was 7.71, 8.04 and 7.60 in lakes, wetlands and rivers, respectively. The range was 6.78 to 8.20, 6.95 to 8.54 and 4.56 to 8.94 in lakes, wetlands and rivers, respectively. The TN ranged from 0.06% to 0.98%, 0.04% to 0.49% and 0.01% to 0.61% and the average concentration was 0.21%, 0.19% and 0.11% in lakes, wetlands and rivers, respectively. The average content of TC ranged from 0.56% to 13.62%, 0.82% to 6.00% and 0.12% to 7.78% in lakes, wetlands and rivers, respectively. The order of the averaged concentration of TC was 2.66% (lakes sediment), 2.58% (wetland sediments) and 1.42% (river sediments). The ratio of C/N ranged from 6 to 20, 10 to 42 and 6 to 107 in lakes, wetlands and rivers, respectively. The high and low were in river (20) and lake (11) sediments (S2 Table).

## Discussion

### Excessive content of P in surface sediments

**Fig 3:**
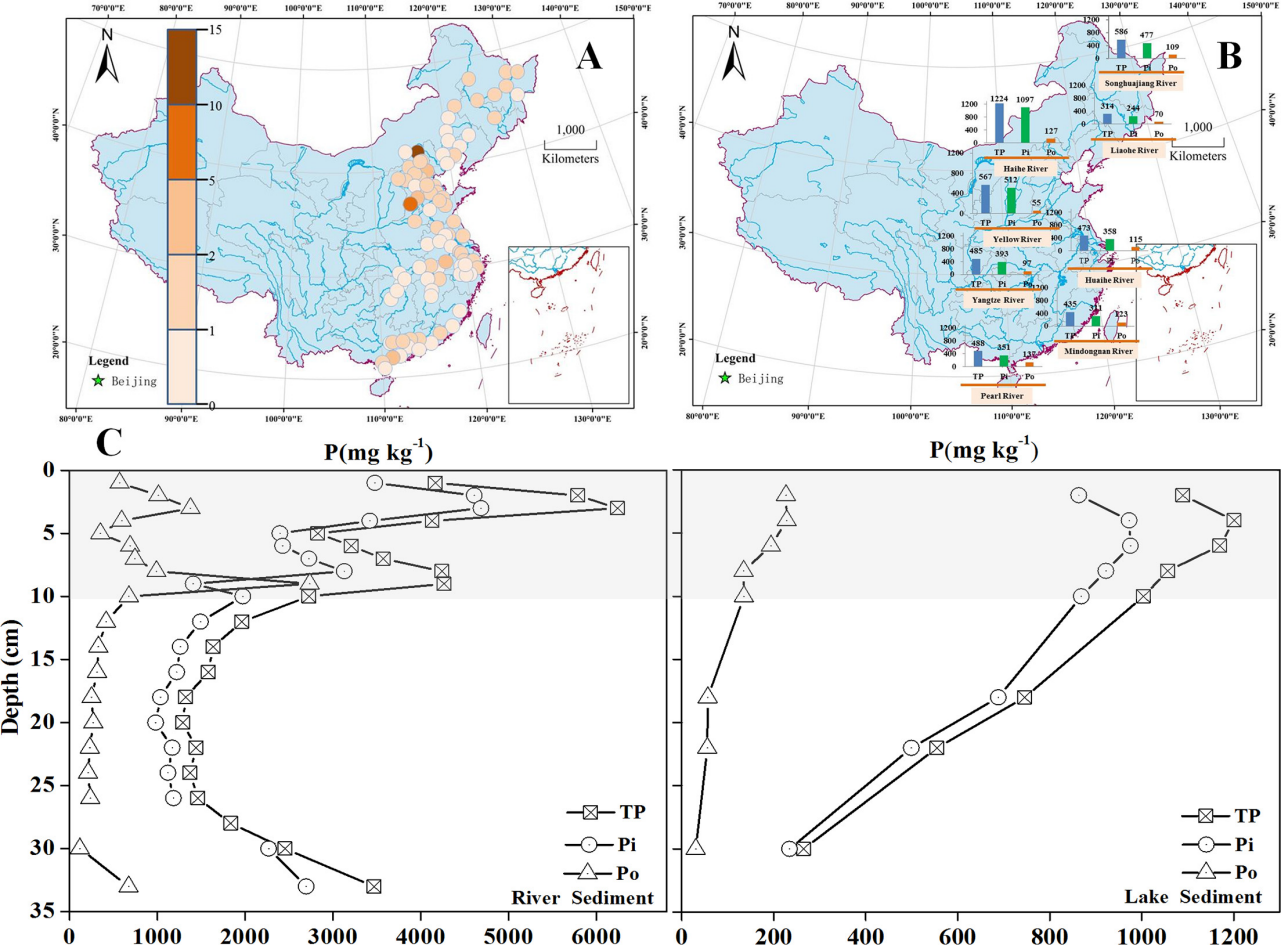
The distribution of P in freshwater ecosystems of Eastern China. A: TP concentration in surface sediment, where the coefficient is P content per 500 mg kg^−1^ [28]. B: Average concentrations of TP, Pi and Po in different river systems. C: TP, Pi and Po concentrations in river and lake sediment cores in Eastern China.

More than 50% of the 89 sampling sites had P concentrations exceeding 500 mg kg^−1^, indicating P pollution in the surface sediments [28]. When external P inflow decreases in these freshwater ecosystems, the sediments can become a source of P (Fig 3A). The surface sediment P concentration varied among watersheds (Fig 3B). At 1224.4 and 1097.1 mg kg^−1^, the highest concentrations of TP and Pi were in the Haihe River system. Concentrations of TP were 314.0, 567.5 and 486.1 mg kg^−1^ in the Liaohe River, Yellow River and Yangtze River systems, respectively, and Pi concentrations were 243.6, 512.2 and 353.5 mg kg^−1^. High P concentrations in the Haihe River system were driven by sewage inputs to the watershed. Population growth and economic development increase sewage production and water scarcity, threatening the health of the Haihe River system [9]. Moreover, dams intensify P accumulation in the sediment. Beiyunhe River, an important component of the Haihe River system, has a dozen of dams in a ~100 km stretch. Numerous dams in Haihe River system slow down the flow, dividing the river into many reservoirs [29].

### Pi was the primary driver of TP variation in different river systems

Pi was the primary driver of variation in TP among different river systems (Fig 3B). Pi ranged from 71% to 90% of TP, with the largest percentage in the Yellow River (90%) and Haihe River (89%) systems. HCl-Pi concentrations ranged from 0.6 to 15.6 times than NaOH-Pi, with the maximum concentration (15.6) found in the surface sediments of the Yellow River system. With the highest suspended sediment concentration in the world (22–65 g L^−1^), and Ca concentrations ranging from 60 to 80 mg L^−1^ in the lower reaches, HCl-Pi is the major form of P in the sediment of the Yellow River [30]. We found that Po accounted for 20% of TP in the surface sediment, which ranged from 9.7% to 28.4%. Mono-P was the major form of Po in the sediment and ranged from 68.3% to 94.2% in different river systems. Po can act as an important source of P in limnetic ecosystems through mineralization to Pi, which is bioavailable and can be recycled to surface waters [31,32]. We also found that P mainly existed in sediments less than ~10 cm deep. Samples collected from Fuyanghe River and Chaohu Lake showed that the TP, Pi and Po concentrations dramatically decreased below 10 cm (Fig 3C). The surface sediment is an important part of the P biogeochemical cycle, as it is where transformation or mineralization of P speciation primarily occurs.

### Po originate from both endogenetic and anthropogenic source

Po was related to TC and TN, indicating that Po may originate from both endogenetic and anthropogenic source. Researchers indicated that the ratio of TC to TN was a significant index of OM origin [33]. The ratio of TC to TN ranged from 2.6 to 4.3 in bacteria, and 7.7 to 10.1 in algae. But the ratio was higher than 10 due to the lack of protein and rich of cellulose in higher vascular plants, such as terrestrial plant and helophyte [34,35]. The sampling sites which the ratio of TC to TN were higher than 14 and lower than 10 account for 17.9% and 35.9% of total sampling sites, respectively. The sampling sites which the ratio of TC to TN ranging from 10 to 14 accounts for nearly 50% of total sampling sites, which means the OM originated from endogenetic and anthropogenic source. The Po and LOI had positive relationship (R^2^ = 0.54, *p*<0.001), which indicated OM and Po have the same origin. This study proved that the Po had complex origin, which included endogenetic and anthropogenic sources.

### OM and pH affect the speciation of P

**Fig 4:**
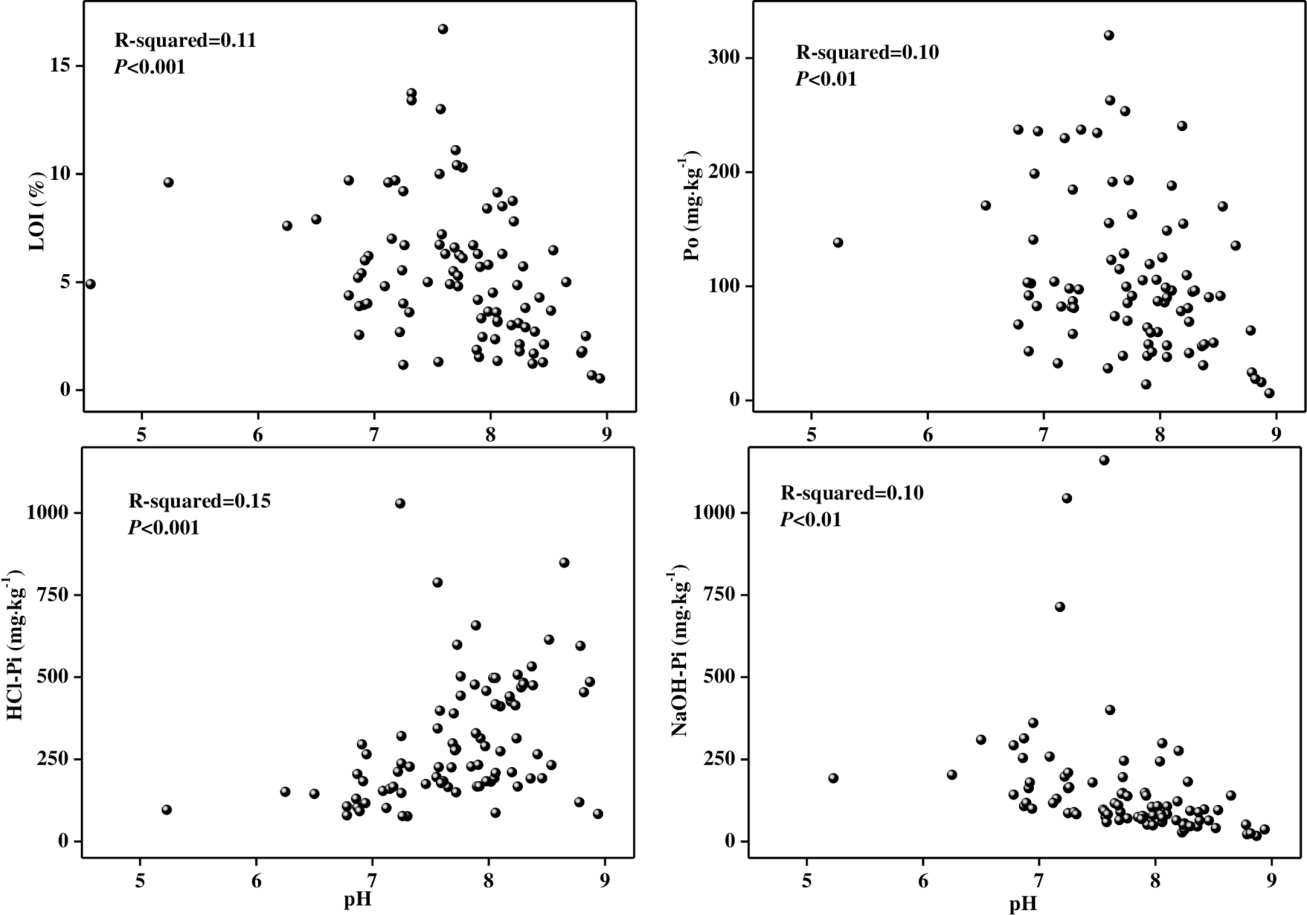
Relationships between pH and NaOH-Pi, HCl-Pi, LOI and Po in sediments of Eastern China. NaOH-Pi, LOI and MUP were negatively correlated with pH (*p* < 0.01, n = 89); HCl-Pi was positively correlated with pH (*p* < 0.01, n = 89).

**Fig 5:**
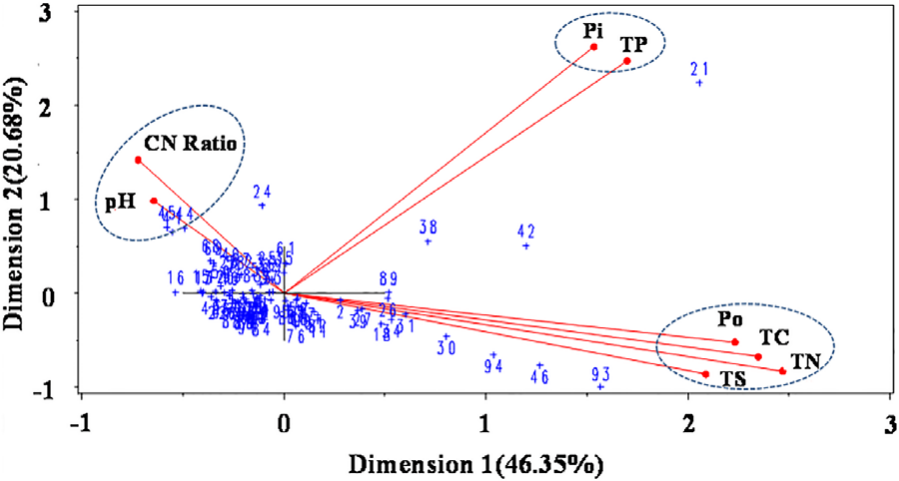
Biplot of sediment properties and sediment P among sample sites.

At the regional scale, NaOH-Pi, HCl-Pi and Po were differently related to sediment pH, with negative relationships for NaOH-Pi and Po and a positive correlation for HCl-Pi (all *p* < 0.01, n = 89) (Fig 4). These varying relationships demonstrate that sediment pH can affect the forms in which P can exist, where NaOH-Pi and Po are more labile than HCl-Pi in sediments [36,37]. As pH decreased, the concentrations of NaOH-Pi and Po increased, but HCl-Pi decreased. These results suggest that P bioavailability will increase if sediment pH decreases. Thus, in freshwater systems of Eastern China, eutrophication of rivers and lakes is likely continue with the decrease of pH, even if external P inputs are reduced. The biplot of P and sediment properties is shown in Fig 5. The first and second principal components accounted for 46.35% and 20.68% of the variation, respectively, indicating a better fitting level [38]. The angles between environment variables indicated that TP and Pi are related, where Pi is an important determinate of TP concentration.

The apparent relationship between pH and the ratio of TC to TN suggested both OM origin and pH may control the existence of P forms. From above analysis, the ratio of TC to TN will affect the origin of OM. Large amounts of wastewater input lead to accumulation of P in the sediment, due to the rapid socio-economic development in Eastern China. The complex composition of man-made pollutant input will affect the composition of Po in sediment. For the sediment rich in aquatic plant, most of Po originated from decomposition of aquatic plants, such as algae, and the composition of Po was relatively simple. Turner et al suggested that higher adsorptive surface area and lower microbial activity were beneficial for the existence of Po in low pH acid-soil. On the contrary, the alkaline soil, which was mainly characterized by CaCO_3_, had a worse ability for holding organic carbon and Po [39]. For most regions in eastern China, the complicated sediment characteristic will affect the Po speciation.

Many researches indicate pH can affect the existing of P forms by influencing the combination of P and different ions. The dominating forms are Fe/Al-P under the condition of decreasing or low pH. Carbonate can adsorb phosphate when the CO_3_^2-^ content increasing as the pH arising [40–44]. The Fe^3+^ has the dominated sorption capacities of P. But the P will be released during the development of hypolimnetic anoxia that results in the seductive dissolution of Fe^3+^ solid phases and release of Fe^2+^ into solution or reprecipitation as Fe^2+^ sulfide [45,46]. Al^3+^ has been proved to prevent P release during anoxia by adsorbing the P liberated from Fe^3+^[47,48]. The Al^3+^ has a high sorption capacity, provided that sediment pH is circumneutral [49]. Such pH conditions are produced by microbial reduction that generates alkalinity by the carbonate and sulfide buffering systems. Recent study has demonstrated that the P forms will pH affected, which can be associated with hydrogen sulfide oxidation in deep layers coupled with oxygen reduction present in top layer [50]. This produce spatially separated was conducted by electroactive bacterial, which was called “cable bacteria” [50,51].

### The characteristic of P speciation in different ecosystems

The physicochemical properties of sediments from the different limnetic ecosystems are shown in Table 1. The pH levels in lake and wetland sediments were neutral, ranging 6.78–8.54. The pH of riverine sediments ranged widely from 4.56 to 8.94. Sewage inflow may have caused the low pH in river sediments. As above analysis, the ratio of TC to TN indicated the OM origin [52,53]. In the lake sediments, low ratio of TC to TN (mean: 11; range: 6–20) indicated that OM content mostly originated from the decomposition of aquatic plants. In wetland sediments, ratio of TC to TN ranged from 10 to 42, pointing to an OM origin from both algae and vascular plants. Mean ratio of TC to TN exceeded 20 in river sediments (range: 6–107), suggesting a primarily anthropogenic OM origin. Different sources of OM resulted in the variation in concentrations of P speciation in the lake, wetland and river sediments.

**Table 1:** Sediment characteristics in different freshwater ecosystems in Eastern China.

Sample		TP (mg kg^-1^)	Po (mg kg^-1^)	TN (%)	TC (%)	TC/TN	pH
Lakes	M	425	121	0.21	2.66	11	7.71
Lakes	R	269–641	49–262	0.06–0.98	0.56–13.62	6–20	6.78–8.20
Wetlands	M	599	128	0.19	2.58	17	8.04
Wetlands	R	379–903	42–253	0.04–0.49	0.82–6.00	10–42	6.95–8.54
Rivers	M	758	99	0.11	1.42	20	7.60
Rivers	R	87–6124	6–319	0.01–0.61	0.12–7.78	6–107	4.56–8.94

M: mean value, R: range value

**Fig 6:**
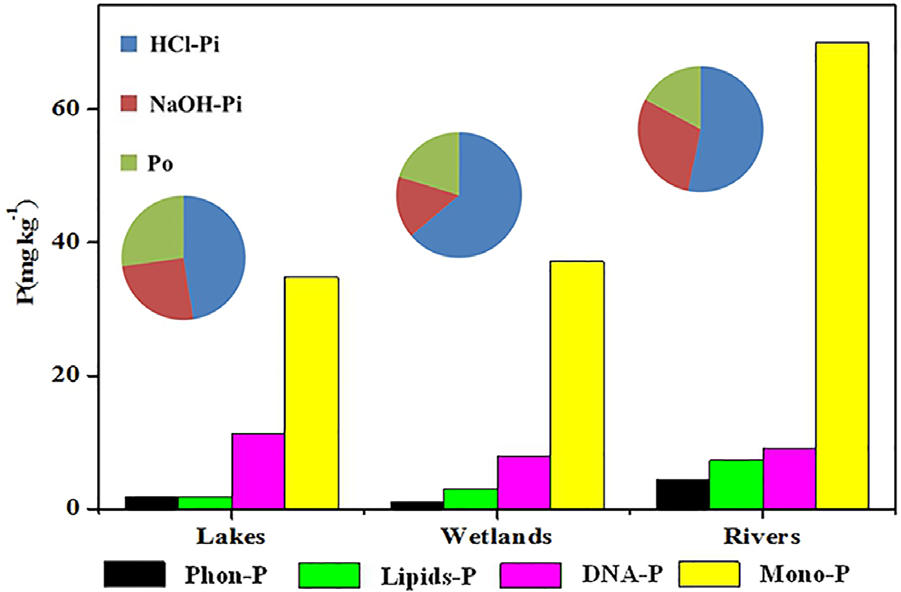
P components in surface sediment determined by SMT fractionation and ^31^P-NMR. DNA-P: deoxyribonucleic acids (orthophosphate diesters); Lipids-P: phospholipids (orthophosphate diesters); Mono-P: orthophosphate monoesters; Ortho-P: orthophosphate; Phon-P: phosphonates; Pyro-P: pyrophosphates.

Sediment P concentrations varied widely among ecosystem types (Fig 6). TP concentrations were generally higher in river sediments (87–6124 mg kg^−1^) than in lake or wetland sediments (Table 1). The terrain in Eastern China drives river flow from west to east, and the plains slow the flow. Large cities are located along the rivers, yet environmental legislation and sewage treatment has not kept pace with rapid economic and population growth. Therefore, large amounts of untreated wastewater and rubbish have been directly discharged into the environment. Consequently, large P loads are being carried into the rivers [4,54]. The riverine sampling sites in this study were located in the flood plain, where the construction of dams and irrigation channels has significantly slowed the main water body. For example, there are 18 dams over a 100 kilometer stretch in the Beiyunhe River in the Haihe River Basin [9,28,55]. Together, the dams and P inflow result in the accumulation of P on the sediments, such as in Fuyanghe River, where TP reached 6000 mg kg^−1^. Mean P concentrations in the wetlands and lakes (425.75 and 599.38 mg kg^−1^, respectively) were lower than those in the rivers.

The concentration of HCl-Pi was higher in wetland sediments than that in riverine or lake sediments. Conversely, NaOH-Pi was the lowest in wetland sediments. Variation in water level can affect redox conditions, and the interaction of oceanic and fresh water in coastal wetlands may affect the transformation of different forms of P in the sediment, resulting in the observed patterns. P in wetlands was bound to Ca/Mg and existed as apatite. Wetlands are an important P-pool in the coastal zone [56]. In contrast, Po concentrations were higher in lake sediments than in wetland or riverine sediments, and lakes acted as P sinks. Thus, anthropogenic P inflow to lakes through rivers and storm runoff will accumulate in lake ecosystems as OM formed by primary production [57].

Three classes of Po compounds and one Po compound were detected in sediments using ^31^P-NMR: Phon-P, Mono-P, Lipids-P and DNA-P (Fig 2). Concentration of different Po compounds as similar as the previously results, such as Taihu Lake and Chaohu Lake [11,32, 58–60]. Biogenic P compounds include Po as well as Poly-P and Pyro-P, which were analyzed as the non-molybdenum-reactive P in a NaOH extract [22, 31]. As important P components, the average concentrations of biogenic P were different in wetlands, lakes and rivers, which were 46.1, 48.4 and 77.0 mg kg^-1^, respectively. We deduced that the wastewater input into the river systems, which carry large anthropogenic pollution, was the principal reason for high biogenic P in rivers. The half-life times of the biogenic P were about 13 yr for Pyro-P, 21 yr for Diester-P and 23 yr for Mono-P, respectively in Lake Erken (Swenden) and 3 yr for Pyro-P, 12 yr for DNA-P, 14 yr for Lipids-P and 27 yr for Mono-P, respectively in Lake Taihu (China) [22,59]. Studies indicated the biogenic P was mineralized to Ortho-P and potentially available recycled into the surface water and supported further phytoplankton growing, and induced the eutrophication [11,31,32].

The concentrations of these four Po species differed among wetlands, rivers and lakes. Total Po was the lowest in riverine sediments, but Mono-P was the highest, with the average concentration of 48 mg kg^−1^. Mono-P represents a wide range of important Po compound classes, including inositol phosphate and sugar phosphates, and has a long half-life in the sediment (23 years). Rivers act as the corridors of watersheds, receiving the majority of anthropogenic pollution. However, the relatively stable HCl-Pi and Mono-P component classes that were the dominant P compounds do not easily cause eutrophication in flowing rivers. In the Haihe River system, in contrast, overuse of water resources, environmental deterioration and numerous dams that slow flow may cause Mono-P to be mineralized into Ortho-P, resulting in eutrophication [9]. At 27% of TP, sediment Po concentrations were highest in lakes. Diesters-P concentrations were also highest in lakes. Diesters-P, a more labile Po species than Mono-P, is composed of DNA-P, Lipids-P and Teichoic-P compound classes and has a half-life of 21 years [26]. DNA-P, which comes from the decomposition of aquatic organisms, is easily mineralized and affects surface water quality. Higher concentrations of DNA-P in lake and wetland sediments indicated higher rates of primary production than in rivers. Hydrophyte metabolism may have lead to these higher Po concentrations [11, 26].

## Conclusions

The combination of SMT and ^31^P-NMR methods has provided information on P characteristics of sediments from P in rivers, wetlands and lakes in Eastern China. The results indicate that P pollution of the surface sediments was serious. Pi was the primary driver of variation in TP among different river systems, and the Pi ranged from 71% to 90% of TP in surface sediment in Eastern China. Environmental factors affect the speciation and origin of P. Po originates from both endogenetic and anthropogenic source. OM and pH affect the speciation of P. The physicochemical properties of sediments from the different limnetic ecosystems affect the P speciation.

## Supporting Information

S1 Fig Part of photographs of the sediment sample sites. (DOCX)

S1 Table Location of the sample sites in Eastern China. (DOCX)

S2 Table The data for individual samples. (DOCX)
