# Supplementary figures and images for: Characteristics and Distribution of Phosphorus in Surface Sediments of Limnetic Ecosystem in Eastern China
Source: PLoS One. 2016 Jun 9;11(6):e0156488. doi: 10.1371/journal.pone.0156488 (PMC4900520; doi:10.1371/journal.pone.0156488)

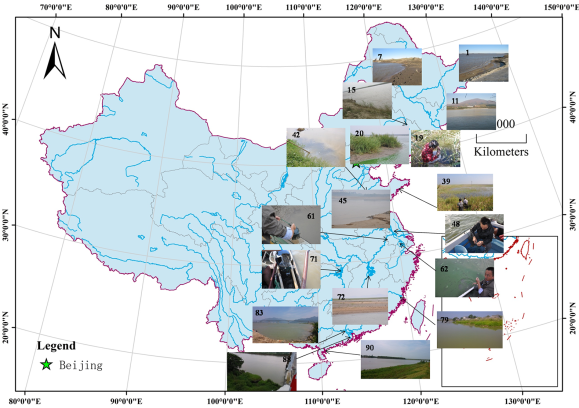


**S1 Figure.** Part of photographs of the sediment sample sites

Supplement: S1 Fig — (DOCX) [file pone.0156488.s001.docx]
