# Supplementary material for: Characteristics and Distribution of Phosphorus in Surface Sediments of Limnetic Ecosystem in Eastern China
Source: PLoS One. 2016 Jun 9;11(6):e0156488. doi: 10.1371/journal.pone.0156488 (PMC4900520; doi:10.1371/journal.pone.0156488)
Supplement: S1 Table — (DOCX) [file pone.0156488.s002.docx]

**S1 Table.** Location of the sample sites in Eastern China

| Sites | Location | | River System | Classification |
| --- | --- | --- | --- | --- |
|  | N | E |  |  |
| 1 | 47°42'27.00" | 132°32'43.00" | Songhuajiang | Wetland（Sanjiang Wetland） |
| 2 | 47°40'55.00" | 132°31'22.00" |  |  |
| 3 | 47°14'49.00" | 132° 0'16.00" |  | River |
| 4 | 46°48'47.00" | 130°12'15.00" |  | River |
| 5 | 46°17'50.00" | 129°32'57.00" |  | River |
| 6 | 45°20'36.00" | 132°17'19.00" |  | Lake(Xingkaihu Lake) |
| 7 | 47°15'58.00" | 123°51'53.00" |  | River |
| 8 | 45°46'45.00" | 126°28'27.00" |  | River |
| **9** | **45°13'41.56"** | **124°16'59.67"** |  | **/** |
| 10 | 45° 9'18.00" | 124°47'55.00" |  | River |
| 11 | 43°44'26.00" | 126°40'11.00" |  | River |
| 12 | 43°31'33.00" | 123°31'9.00" | Liaohe | River |
| 13 | 43°25'16.00" | 123°43'14.00" |  | River |
| **14** | **43°30'50.97"** | **122° 4'3.82"** |  | **/** |
| 15 | 42°18'28.00" | 123°38'49.00" |  | River |
| 16 | 41°57'43.30" | 122°46'6.18" |  | River |
| 17 | 41°11'10.99" | 122° 5'48.06" |  | River |
| 18 | 40°59'58.00" | 121°48'24.00" |  | Wetland(Liaohekou Wetland) |
| 19 | 41° 1'59.00" | 121°53'23.00" |  |  |
| 20 | 41° 1'23.00" | 121°48'22.00" |  |  |
| 21 | 42°11'18.00" | 119°14'26.00" | Haihe | River |
| 22 | 42°12'21.00" | 116°38'14.00" |  | River |
| 23 | 40°45'3.00" | 118° 8'23.00" |  | River |
| 24 | 39°44'41.00" | 118°45'45.00" |  | River |
| 25 | 39°42'52.00" | 116°56'34.00" |  | River |
| 26 | 39°15'17.00" | 117° 5'26.00" |  | River |
| 27 | 39°16'43.00" | 117°33'14.00" |  | Wetlanf(Qilihai Wetland) |
| 28 | 39°18'33.00" | 117°33'43.00" |  |  |
| 29 | 39° 6'57.00" | 117°42'52.00" |  | River |
| 30 | 38°55'53.00" | 115°59'53.00" |  | Wetland(Baiyangdian Wetland) |
| 31 | 38°54'29.00" | 115°57'51.00" |  |  |
| 32 | 38°51'39.00" | 116° 4'44.00" |  |  |
| 33 | 38°44'37.39" | 117°16'13.04" |  | Wetland(Beidagang Wetland) |
| 34 | 38°45'54.00" | 117°22'58.00" |  |  |
| 35 | 38°36'37.00" | 117°19'9.00" |  | River |
| 36 | 38°31'12.00" | 117°27'52.00" |  | Wetland(Nandagang Wetland) |
| 37 | 38°30'31.00" | 117°28'7.00" |  |  |
| 38 | 38°10'13.00" | 116° 4'43.00" |  | River |
| 42 | 37°30'3.00" | 115° 3'27.00" |  | River |
| **43** | **34°54'39.50"** | **113°40'0.60"** | Yelllow River | **/** |
| 39 | 38° 1'3.00" | 118°50'42.00" |  | Wetland(Huanghekou Wetland) |
| 40 | 37°51'36.00" | 119° 4'23.00" |  |  |
| 41 | 37°35'45.00" | 118°32'48.00" |  | River |
| 44 | 35°57'40.00" | 115°54'2.00" |  | River |
| 45 | 36°44'8.00" | 116°56'1.00" |  | River |
| 46 | 35° 4'57.00" | 116°49'19.00" | Huaihe | Lake(Weishanhu Lake) |
| 47 | 34°40'30.57" | 117°10'44.19" |  |  |
| 48 | 34° 4'8.00" | 118°12'14.00" |  | Lake(Luomahu Lake) |
| 49 | 32°27'8.00" | 116°15'47.00" |  | River |
| 50 | 32°57'45.00" | 117°25'41.00" |  | River |
| 51 | 33°11'56.00" | 118°23'40.00" |  | Lake(Hongzehu Lake) |
| 52 | 33°21'0.00" | 118°47'17.00" |  |  |
| 53 | 33°56'25.00" | 119°49'2.00" |  | River |
| 54 | 32°46'50.00" | 119°13'44.00" |  | River |
| 55 | 32°49'42.00" | 119°20'17.00" |  | River |
| 56 | 31°55'38.00" | 120°15'13.00" | Yangtze River | River |
| 57 | 31°50'8.00" | 121°18'28.00" |  | River |
| 58 | 31°49'25.00" | 121°23'33.00" |  | River |
| 59 | 31°36'54.00" | 117°22'17.00" |  | Lake(Chaohu Lake) |
| 60 | 31°33'51.00" | 117°37'24.00" |  |  |
| 61 | 31°42'40.00" | 118°27'4.00" |  | River |
| 62 | 31°29'30.00" | 120°11'6.00" |  | Lake(Taihu Lake) |
| 63 | 31°25'17.00" | 120°10'4.00" |  |  |
| 64 | 31°17'33.00" | 120° 7'45.00" |  |  |
| 65 | 31° 6'23.00" | 120° 5'49.00" |  |  |
| 66 | 30°59'15.00" | 120°19'14.00" |  |  |
| 67 | 30°30'8.00" | 117° 8'38.00" |  | River |
| 68 | 30°15'37.66" | 115° 4'46.00" |  | River |
| 69 | 29°33'41.00" | 113°13'4.00" |  | River |
| 70 | 29°14'15.00" | 113° 0'25.00" |  | Lake(Dongtinghu Lake) |
| 71 | 28°48'19.00" | 112°44'27.00" |  |  |
| 72 | 29°36'51.00" | 116° 9'6.00" |  | Lake(Poyanghu Lake) |
| 73 | 29°10'22.00" | 116° 3'1.00" |  |  |
| 74 | 29° 3'3.00" | 116°10'23.00" |  |  |
| 93 | 29°47'37.00" | 113°18'58.00" |  | Lake(Honghu Lake) |
| 94 | 29°53'53.00" | 113°23'40.00" |  |  |
| **75** | **27° 7'21.97"** | **115° 0'15.56"** |  | **/** |
| 76 | 26° 9'34.00" | 119° 5'26.00" | Mindongnan | River |
| 77 | 24°56'52.00" | 118°30'55.00" |  | River |
| 78 | 24°31'9.00" | 117°46'60.00" |  | River |
| 79 | 23°41'26.00" | 116°38'43.00" |  | River |
| 80 | 23°41'43.65" | 113° 5'46.16" | Pearl River | River |
| 81 | 23°36'15.65" | 114°40'34.75" |  | River |
| 82 | 23°31'27.22" | 113°33'41.23" |  | River |
| 83 | 23° 6'35.90" | 113°53'56.41" |  | River |
| 84 | 22°47'58.97" | 113°36'57.23" |  | River |
| 85 | 22°34'6.12" | 113°10'36.33" |  | River |
| 86 | 23° 3'37.81" | 112°31'18.36" |  | River |
| 87 | 23° 5'21.36" | 111°58'56.23" |  | River |
| 88 | 21°30'58.81" | 110°43'38.00" |  | River |
| 89 | 21°23'42.95" | 110°23'11.02" |  | River |
| 90 | 21° 6'32.09" | 110°12'4.06" |  | River |
| 91 | 20°52'32.13" | 110° 3'29.73" |  | River |
| **92** | **19°58'21.73"** | **110°24'39.65"** |  | **/** |
