# Supplementary material for: Characteristics and Distribution of Phosphorus in Surface Sediments of Limnetic Ecosystem in Eastern China
Source: PLoS One. 2016 Jun 9;11(6):e0156488. doi: 10.1371/journal.pone.0156488 (PMC4900520; doi:10.1371/journal.pone.0156488)
Supplement: S2 Table — (DOCX) [file pone.0156488.s003.docx]

**S2 Table.** The data for individual samples

| **Watershed** | **Sample sites** | **N%** | **C%** | **C/N** | **pH** | **LOI%** | **PO** | **NaOH-P** | **HCl-P** | **TP** |
| --- | --- | --- | --- | --- | --- | --- | --- | --- | --- | --- |
|  |  |  |  |  |  |  | **mg kg^-1^** | | | |
| Songhuajiang | 1 | 0.12 | 1.24 | 10.74 | 7.09 | 4.80 | 103.97 | 258.22 | 153.60 | 515.79 |
|  | 2 | 0.21 | 2.24 | 10.46 | 6.95 | 6.20 | 235.80 | 360.56 | 264.99 | 861.35 |
|  | 3 | 0.09 | 1.03 | 10.80 | 6.87 | 2.55 | 91.87 | 313.28 | 204.65 | 609.80 |
|  | 4 | 0.13 | 1.47 | 11.06 | 6.25 | 7.60 |  | 202.89 | 150.58 | 353.47 |
|  | 5 | 0.13 | 1.70 | 12.75 | 6.91 | 3.94 | 140.87 | 161.80 | 295.19 | 597.86 |
|  | 6 | 0.06 | 0.56 | 9.29 | 7.90 | 1.53 | 49.00 | 75.14 | 167.05 | 291.19 |
|  | 7 | 0.10 | 1.19 | 11.43 | 7.22 | 2.68 | 97.99 | 197.34 | 211.97 | 507.30 |
|  | 8 | 0.08 | 0.72 | 9.33 | 7.72 | 4.80 | 85.12 | 146.98 | 281.36 | 513.45 |
|  | 9 |  |  |  |  |  |  |  |  | 0.00 |
|  | 10 | 0.04 | 0.44 | 11.40 | 7.25 | 1.17 | 58.18 | 86.20 | 320.59 | 464.97 |
|  | 11 | 0.17 | 1.60 | 9.54 | 4.56 | 4.90 | 150.52 | 159.82 | 400.91 | 711.26 |
|  | | | | | | | | | | |
| Liaohe | 12 | 0.06 | 1.10 | 17.88 | 8.46 | 2.11 | 50.53 | 63.65 | 192.12 | 306.30 |
|  | 13 | 0.03 | 0.38 | 11.92 | 8.78 | 1.72 | 61.25 | 50.99 | 119.39 | 231.63 |
|  | 14 |  |  |  |  |  |  |  |  | 0.00 |
|  | 15 | 0.02 | 0.32 | 15.15 | 8.36 | 1.22 | 47.47 | 45.21 | 191.44 | 284.11 |
|  | 16 | 0.01 | 0.12 | 12.86 | 8.94 | 0.54 | 6.12 | 36.49 | 83.50 | 126.11 |
|  | 17 | 0.03 | 0.49 | 16.00 | 8.25 | 2.11 | 41.34 | 55.29 | 167.05 | 263.69 |
|  | 18 | 0.20 | 2.59 | 12.98 | 8.54 | 6.47 | 169.96 | 95.25 | 232.06 | 497.27 |
|  | 19 | 0.13 | 1.45 | 11.12 | 8.10 | 6.30 | 96.21 | 106.47 | 274.44 | 477.12 |
|  | 20 | 0.11 | 1.41 | 12.85 | 8.42 | 4.28 | 90.34 | 97.07 | 265.23 | 452.65 |
|  | | | | | | | | | | |
| Haihe | 21 | 0.48 | 7.78 | 16.32 | 7.32 | 13.73 | 306.23 | 1430.30 | 4344.38 | 6080.92 |
|  | 22 | 0.08 | 0.65 | 8.27 | 8.06 | 1.35 | 47.92 | 66.08 | 87.20 | 201.20 |
|  | 23 | 0.12 | 1.61 | 13.38 | 7.89 | 4.17 | 38.86 | 72.11 | 657.60 | 768.57 |
|  | 24 | 0.02 | 0.47 | 25.44 | 8.45 | 1.28 | 15.31 | 172.03 | 1937.18 | 2124.52 |
|  | 25 | 0.11 | 1.13 | 10.59 | 8.04 | 2.35 | 85.75 | 243.30 | 497.11 | 826.15 |
|  | 26 | 0.33 | 3.34 | 10.08 | 7.73 | 6.25 | 192.97 | 245.39 | 598.42 | 1036.78 |
|  | 27 | 0.35 | 4.28 | 12.18 | 8.10 | 8.51 | 188.18 | 82.75 | 410.79 | 681.72 |
|  | 28 | 0.06 | 1.23 | 19.93 | 8.38 | 2.70 | 48.96 | 63.98 | 475.02 | 587.96 |
|  | 29 | 0.10 | 1.98 | 19.89 | 8.28 | 5.72 | 95.19 | 181.68 | 468.91 | 745.77 |
|  | 30 | 0.49 | 6.00 | 12.32 | 7.70 | 11.10 | 253.34 | 89.76 | 389.53 | 732.63 |
|  | 31 | 0.42 | 4.31 | 10.38 | 8.19 | 8.75 | 240.53 | 122.28 | 425.62 | 788.42 |
|  | 32 | 0.06 | 0.82 | 12.80 | 7.93 | 2.46 | 42.36 | 51.12 | 313.28 | 406.75 |
|  | 33 | 0.12 | 2.65 | 22.37 | 7.98 | 3.63 | 59.83 | 79.32 | 458.46 | 597.61 |
|  | 34 | 0.41 | 5.15 | 12.56 | 7.76 | 10.30 | 162.93 | 69.92 | 502.33 | 735.18 |
|  | 35 | 0.13 | 1.95 | 14.70 | 8.06 | 3.15 | 37.83 | 298.66 | 497.11 | 833.59 |
|  | 36 | 0.12 | 2.28 | 19.61 | 8.52 | 3.67 | 91.56 | 40.67 | 614.09 | 746.32 |
|  | 37 | 0.14 | 1.98 | 13.87 | 8.23 | 4.86 | 109.73 | 27.42 | 413.76 | 550.91 |
|  | 38 | 0.34 | 2.64 | 7.87 | 7.24 | 5.54 | 81.69 | 1044.16 | 1028.49 | 2154.34 |
|  | 42 | 0.61 | 3.71 | 6.14 | 7.56 | 6.72 | 319.80 | 1160.32 | 788.10 | 2268.23 |
|  | | | | | | | | | | |
| Yellow River | 39 | 0.36 | 4.60 | 12.86 | 8.06 | 9.15 | 148.74 | 59.04 | 417.71 | 625.49 |
|  | 40 | 0.04 | 1.50 | 42.76 | 8.25 | 1.80 | 68.90 | 35.45 | 507.55 | 611.90 |
|  | 41 | 0.01 | 0.88 | 93.13 | 8.87 | 0.69 | 15.93 | 16.78 | 485.57 | 518.28 |
|  | 43 |  |  |  |  |  |  |  |  | 0.00 |
|  | 44 | 0.01 | 1.15 | 91.49 | 8.79 | 1.80 | 24.40 | 21.81 | 595.23 | 641.44 |
|  | 45 | 0.01 | 1.02 | 107.78 | 8.82 | 2.50 | 18.68 | 24.46 | 454.27 | 497.40 |
|  | | | | | | | | | | |
| Huaihe | 46 | 0.60 | 9.64 | 16.04 | 7.57 | 13.00 | 262.98 | 74.85 | 226.02 | 563.85 |
|  | 47 | 0.15 | 3.16 | 20.41 | 7.58 | 7.20 | 123.01 | 59.04 | 397.95 | 580.00 |
|  | 48 | 0.12 | 1.56 | 13.20 | 7.85 | 6.70 | 105.20 | 74.10 | 227.63 | 406.93 |
|  | 49 | 0.04 | 0.39 | 9.87 | 7.55 | 1.30 | 27.93 | 96.59 | 195.39 | 319.91 |
|  | 50 | 0.03 | 0.54 | 16.59 | 8.24 | 3.10 | 80.69 | 50.99 | 313.55 | 445.23 |
|  | 51 | 0.24 | 3.12 | 12.84 | 7.69 | 6.60 | 128.70 | 64.97 | 298.15 | 491.82 |
|  | 52 | 0.15 | 2.71 | 18.41 | 7.56 | 10.00 | 155.32 | 92.63 | 343.61 | 591.55 |
|  | 53 | 0.06 | 0.84 | 14.32 | 7.89 | 6.30 | 63.72 | 77.81 | 328.78 | 470.32 |
|  | 54 | 0.12 | 1.24 | 10.03 | 7.71 | 10.40 | 99.58 | 145.12 | 276.72 | 521.43 |
|  | 55 | 0.11 | 1.20 | 11.37 | 7.97 | 8.40 | 105.52 | 105.31 | 289.41 | 500.23 |
|  | | | | | | | | | | |
| Yangtze River | 93 | 0.98 | 13.62 | 13.96 | 7.59 | 16.70 | 191.48 | 82.45 | 177.50 | 451.43 |
|  | 94 | 0.69 | 9.89 | 14.33 | 7.32 | 13.40 | 237.14 | 82.75 | 227.01 | 546.90 |
|  | 56 | 0.10 | 1.87 | 18.96 | 7.76 | 6.10 | 91.55 | 138.08 | 443.40 | 673.04 |
|  | 57 | 0.04 | 1.29 | 32.75 | 8.30 | 2.90 |  | 45.96 | 482.56 | 528.51 |
|  | 58 | 0.05 | 1.46 | 28.50 | 8.18 | 3.00 | 78.06 | 64.06 | 441.31 | 583.44 |
|  | 59 | 0.23 | 1.55 | 6.63 | 8.20 | 7.80 | 154.65 | 275.43 | 210.21 | 640.30 |
|  | 60 | 0.13 | 0.89 | 6.85 | 7.65 | 4.90 | 114.83 | 116.38 | 165.67 | 396.87 |
|  | 61 | 0.10 | 2.13 | 21.27 | 8.65 | 5.00 | 135.51 | 139.24 | 848.46 | 1123.21 |
|  | 62 | 0.13 | 0.96 | 7.23 | 7.91 | 5.70 | 119.30 | 148.25 | 232.85 | 500.41 |
|  | 63 | 0.12 | 0.90 | 7.23 | 8.02 | 4.50 | 125.20 | 107.32 | 181.76 | 414.28 |
|  | 64 | 0.09 | 0.76 | 8.03 | 8.05 | 3.60 | 98.68 | 82.17 | 191.82 | 372.68 |
|  | 65 | 0.07 | 0.59 | 8.25 | 8.06 | 3.20 | 90.00 | 71.88 | 208.24 | 370.12 |
|  | 66 | 0.15 | 1.49 | 10.15 | 7.98 | 5.80 | 86.90 | 48.97 | 182.77 | 318.65 |
|  | 67 | 0.05 | 1.49 | 27.12 | 8.30 | 3.80 | 96.21 | 93.24 | 477.53 | 666.97 |
|  | 68 | 0.03 | 1.42 | 45.22 | 8.37 | 1.69 | 30.62 | 88.72 | 532.62 | 651.96 |
|  | 69 | 0.04 | 1.80 | 50.80 | 7.88 | 1.86 | 13.78 | 67.93 | 477.00 | 558.71 |
|  | 70 | 0.11 | 0.91 | 8.43 | 7.68 | 5.50 | 38.82 | 110.34 | 225.02 | 374.18 |
|  | 71 | 0.08 | 0.83 | 10.65 | 7.92 | 3.32 | 59.30 | 139.51 | 167.68 | 366.49 |
|  | 72 | 0.11 | 1.01 | 9.48 | 7.72 | 5.28 | 69.57 | 195.85 | 149.57 | 414.99 |
|  | 73 | 0.07 | 0.77 | 10.66 | 6.87 | 3.89 | 42.96 | 107.45 | 103.50 | 253.92 |
|  | 74 | 0.13 | 1.11 | 8.74 | 6.78 | 4.38 | 66.42 | 142.04 | 78.80 | 287.26 |
|  | 75 |  |  |  |  |  |  |  |  |  |
|  | | | | | | | | | | |
| Mindongnan | 76 | 0.14 | 2.00 | 14.34 | 5.23 | 9.60 | 138.11 | 191.82 | 96.26 | 426.19 |
|  | 77 | 0.06 | 0.85 | 14.18 | 7.15 | 7.00 | 82.24 | 129.45 | 159.63 | 371.33 |
|  | 78 | 0.11 | 1.60 | 14.57 | 6.50 | 7.90 | 170.69 | 309.53 | 144.54 | 624.76 |
|  | 79 | 0.07 | 0.88 | 12.31 | 6.89 | 5.40 | 102.42 | 117.34 | 91.65 | 311.40 |
|  | | | | | | | | | | |
| Pearl River | 80 | 0.08 | 1.03 | 12.77 | 6.94 | 4.00 | 82.48 | 99.55 | 116.35 | 298.38 |
|  | 81 | 0.09 | 0.89 | 10.17 | 6.86 | 5.20 | 103.10 | 254.20 | 129.45 | 486.75 |
|  | 82 | 0.16 | 1.82 | 11.40 | 6.78 | 9.70 | 237.14 | 292.23 | 106.47 | 635.83 |
|  | 83 | 0.07 | 0.88 | 12.82 | 7.61 | 6.30 | 73.64 | 399.97 | 182.72 | 656.34 |
|  | 84 | 0.06 | 0.79 | 13.50 | 7.30 | 3.60 | 97.21 | 89.21 | 76.14 | 262.56 |
|  | 85 | 0.07 | 1.04 | 15.42 | 7.25 | 4.00 | 86.90 | 161.83 | 237.03 | 485.76 |
|  | 86 | 0.09 | 1.09 | 11.66 | 6.92 | 6.00 | 198.63 | 179.75 | 182.77 | 561.15 |
|  | 87 | 0.11 | 1.07 | 10.07 | 7.46 | 5.00 | 234.32 | 179.58 | 174.64 | 588.54 |
|  | 88 | 0.07 | 0.86 | 12.80 | 7.26 | 6.70 | 81.01 | 164.96 | 77.23 | 323.20 |
|  | 89 | 0.19 | 2.26 | 11.97 | 7.18 | 9.70 | 229.66 | 713.31 | 166.01 | 1108.98 |
|  | 90 | 0.17 | 1.94 | 11.41 | 7.25 | 9.20 | 184.66 | 208.93 | 147.56 | 541.15 |
|  | 91 | 0.12 | 1.40 | 11.43 | 7.12 | 9.60 | 32.40 | 117.34 | 101.53 | 251.27 |
|  | 92 |  |  |  |  |  |  |  |  |  |
